# Mobile Applications for Participation at the Shopping Mall: Content Analysis and Usability for Persons with Physical Disabilities and Communication or Cognitive Limitations

**DOI:** 10.3390/ijerph111212777

**Published:** 2014-12-10

**Authors:** Claudine Auger, Emilie Leduc, Delphine Labbé, Cassioppée Guay, Brigitte Fillion, Carolina Bottari, Bonnie Swaine

**Affiliations:** 1 Center for Interdisciplinary Research in Rehabilitation of Greater Montreal—Lucie-Bruneau Rehabilitation Center (CRIR-CRLB), 2275 Avenue Laurier East, Montreal, QC H2H 2N8, Canada; E-Mails: emilie.leduc.2@umontreal.ca (E.L.); cassioppee.guay-gallant@umontreal.ca (C.G.); BFillion.crlb@ssss.gouv.qc.ca (B.F.); carolina.bottari@umontreal.ca (C.B.); bonnie.swaine@umontreal.ca (B.S.); 2 School of Rehabilitation, Université de Montréal, C.P. 6128 Succursale Centre-Ville, Montreal, QC H3C 3J7, Canada; 3 Department of Psychology, University of Quebec at Montreal, C.P. 888 Succursale Centre-Ville, Montreal, QC H3C 3P8, Canada; E-Mail: labbe.delphine@courrier.uqam.ca

**Keywords:** universal design, mobile applications, participation, user-centered design, evaluation

## Abstract

The aim of this exploratory study was to determine the important features in content and usability of existing mobile applications evaluating environmental barriers and facilitators (EBF) to participation for persons with physical disabilities presenting mild communication or cognitive limitations. A rigorous process based on a user-centered design approach led to the identification of two relevant mobile applications to evaluate the EBF. An accessibility expert, the research team as well as five users then tested the mobile applications in a shopping mall. A thematic content analysis of the research team’s and users’ comments established 10 categories of key features that adequately respond to the needs of the clientele targeted in this study. In terms of content, granularity and contextualization of the information provided were considered important. With respect to usability, relevant features were place finding, rating system, presentation of results, compatibility, user-friendliness, aesthetics, credibility of the information as well as connectivity/interactiveness. The research team and the users agreed on some aspects such as aesthetics, but had different perspectives on features such as the rating system or the connectivity/interactiveness of the application. The users proposed new features suggesting that the existing mobile applications did not correspond to all their needs.

## 1. Introduction

Shopping can be a gratifying experience allowing stimulating interactions with a physical environment, products, information and other persons [1]. Shopping may also represent a challenge for people with functional limitations because of physical and social barriers. Limited spaces in stores, the presence of obstacles (e.g., stairs, displays), narrow bathrooms and the staff’s attitude (e.g., lack of knowledge about disability, lack of openness) are barriers in shopping malls identified by people living with disabilities, shopkeepers and rehabilitation clinicians [2]. Other factors considered as facilitators include employees’ courtesy, floor configuration, large alleys, adequate access by public or adapted transportation, automatic doors and accessible food courts [2]. Environmental barriers and facilitators (EBF) are thus central to the shopping experience of people living with a disability.

Persons with communication or cognitive limitations can be confronted with significant obstacles when accessing goods and services in the community, thereby affecting their participation in their social roles [3,4]. Stores and restaurants have been identified as places that particularly limit persons with communication limitations [4]. Mazaux and Ricbourg [5] refer to these limitations as an “invisible handicap” which often represent a cause of prejudice and can limit the social and professional reintegration process. Furthermore, there are few facilitators in stores to improve the accessibility of people with communication or cognitive limitations [6]. The prevalence of people with these types of limitations has increased, making the accessibility issue one of central importance [7].

The shopping experience has recently been transformed with the arrival of Internet, smartphones and mobile applications. In 2013, 144.5 million Americans were using smartphones and it is predicted that in 2016, the number of users will increase by more than 50 million [8]. In 2013, nearly 75 % of smartphone owners were using their device as part of their shopping experience, browsing retailer websites (71 %) or using apps (57 %) [9]. Mobile applications are now being developed to evaluate the accessibility of public buildings, and have the potential of fostering interactions between people with physical limitations and their physical and social environment, as well as supporting them in their shopping activities [10,11,12,13].

To date, no systematic review of existing mobile applications designed to evaluate the environment in a shopping context has been completed nor has their content and usability been assessed. Concerning the content of the mobile applications, the social and physical environments should be considered, as well as the users’ needs in terms of communication and cognitive capacities [14]. Concerning usability, understanding ease of use, perceived usefulness and the intention of use by persons with disabilities in a real life context is necessary to analyse the potential for adoption of information technologies [15,16]. Currently available mobile applications should be analysed to determine their ability to optimize the shopping experience of people with mobility, communication or cognitive limitations. The aim of this study was thus to identify the essential features in content and usability of mobile applications evaluating social and physical EBF, with the ultimate goal of ensuring that the needs of the clientele with physical disabilities and mild communication or cognitive limitations are met.

## 2. Methods

### 2.1. Study Design

This study is part of a larger project, the *Rehabilitation Living Lab in the Mall (RehabMaLL)*, whose objective is the creation of an inclusive environment in a shopping mall context for people living with a disability [17]. This is a development study [18], in the context of information technologies, that is based on an iterative user-centered design approach developed by Dabbs [10]. It is comprised of three steps: (1) defining users’ needs to determine the criteria that the application must meet, (2) testing usability iteratively, and (3) redefining tasks to be carried out by the mobile application(s). This paper describes the first two steps.

### 2.2. Step 1: Define Users’ Needs

#### 2.2.1. Procedure

A mobile application is a program downloadable for free or for a minimal fee and executable from the operating system of a mobile device [19]. Between May and August 2013, we identified existing mobile applications designed for evaluating an environment’s EBF using Appstore (Itunes) and GooglePlay search engines. The search was performed using these key words: “access*”, “disabl*”, handicap, “shop*”. Inclusion criteria for the mobile applications were: (1) designed for adults, (2) included an EBF evaluation (e.g., using a rating scale or a EBF checklist), (3) relevant for activities performed at a shopping mall and (4) available in French or English. Exclusion criteria were: (1) the mobile application was geographically restrictive (e.g., evaluated only one city) and (2) the mobile application was specialized for one type of environmental feature only (e.g., bathroom, parking lot).

The research team, whose expertise covers rehabilitation technologies outcome measurement and environmental psychology, assessed the mobile applications. Two student researchers undergoing occupational therapy training completed the research team. Mobile applications were compared using five dimensions based on the team’s judgement: user-friendliness, content, psychometric properties, applicability and other considerations (see Appendix). The content analysis criteria included 144 EBF indicators extracted from Gamache and colleagues [20], Swaine and colleagues [2] and Gauthier and colleagues [6]. These indicators allowed the mapping of the content covered by the mobile applications and comparison of the proportion of indicators covered across the applications. Applicability criteria were based on the work of Auger and colleagues [21]. Each application was analysed on five dimensions regrouping 27 criteria, each rated on a scale from 0 (does not meet the criteria, or the feature is absent) to 2 (completely meets the criteria).

#### 2.2.2. Data Analysis of Step 1

The ratings on each of the five dimensions attributed by the research team members for each application were summed to enable ranking the different applications; the applications in 1st and 2nd positions were retained for testing.

### 2.3. Step 2: Test Usability

#### 2.3.1. Research Team and Accessibility Expert

For this step of the project, the research team was assisted by an accessibility expert from AlterGo, a Montreal-based community organization (Canada) supporting social inclusion in leisure activities for persons with a functional limitation.

#### 2.3.2. Participants (Users)

Participants were persons receiving rehabilitation services from a physical rehabilitation center in Montreal. The goal was to recruit a minimum of five persons with mobility limitations and with mild communication or cognitive limitations. Inclusion criteria were: being over 18 years old, able to consent to the research, presenting a mild communication or cognitive limitation combined with mobility difficulties (needing a technical aid or presence of fatigue) as documented in the participants’ medical record. Furthermore, participants were eligible if they owned a multifunction mobile device (e.g., Android, Apple) or had the knowledge or experience to easily use it. They also had to be able to get to the larger study’s shopping mall by regular or adapted transportation. The shopping mall was an urban complex of three stories located in downtown Montreal visited daily by more than 38,000 visitors [22]. Table 1 describes the characteristics of the sample. All subjects gave their informed consent for inclusion before they participated in the study. The study was conducted in accordance with the Declaration of Helsinki, and the protocol was approved by the Ethics Committee of the Center for Interdisciplinary Research in Rehabilitation of Greater Montreal (CRIR-859-0713).

**Table 1:** Sample characteristics.

No.	Age	Gender	Language Spoken and Understood	Diagnosis	Associated Difficulties	Mobility	Experience with ICT ^1^
1	54	H	FR **^2^** and EN **^3^**	Stroke	Cognitive	Motorized wheelchair and walking	Smartphone
2	26	F	FR and EN	Freidriech’s ataxia	Communication	Manual wheelchair	Smartphone
3	20	H	FR and EN	Freidriech’s ataxia	Communication	Manual and motorized wheelchair	Internet navigation and student in graphic design
4	34	F	FR and help to translate English terms	Steinert myotonic dystrophy	Cognitive	Walking	Smartphone
5	48	H	FR and help to translate English terms	Cerebral palsy	Communication and cognitive	Motorized wheelchair and walking	Smartphone and computer technician

Notes: **^1^** Information and communication technologies; **^2^** French; **^3^** English.

#### 2.3.3. Procedure

The mobile applications were tested in a real environment (authorized by the shopping mall manager), at first by the research team accompanied by an accessibility expert, then with the users to target all aspects of usability [10,23]. Thirteen locations within the mall were selected to cover a range of available services: a bathroom; a restaurant with table service; a restaurant with walk-in service; one supermarket; three stores for house essentials; two smaller stores for clothing and accessories; a store for sports clothing and accessories; a drugstore; a store specialized in small electronic devices; and a shoe store. Accompanied by the two student researchers, the accessibility expert evaluated the stores/locations with the accessibility evaluation grids integrated in the selected mobile applications.

Testing with the users was then conducted in three steps: (1) a pre-shopping semi-structured interview; (2) a one-hour shopping session at the mall with the student researchers and; (3) a post-shopping semi-structured interview. The pre-shopping interview addressed shopping habits, EBFs perceived during usual shopping activities and shopping experience with information and communication technologies. Before they began shopping, participants took a few minutes to manipulate the mobile applications with the help of a student researcher. Then, the users each chose three or four locations from the selected 13. Each store/place visit followed four steps: (1) consulting the available information in the mobile application; (2) visiting the place and accomplishing tasks (e.g., manipulating products, interacting with employees, making a purchase); (3) doing real-time comparison between their own appreciation of the place’s accessibility features and those provided by the mobile application and; (4) modifying the evaluations in the mobile application, if necessary. An electronic tablet (iPad^©^ mini, Apple Inc., Cupertino, CA, USA) was made available to them if they did not own a mobile device. During the shopping session, participants used the *Photo Elicitation* method [24] consisting of using images or videos taken with the mobile device to document his/her experience. During the post-shopping interview, users were asked to share their opinions regarding the mobile applications’ usability by referring to these pictures or videos, and by responding to questions inspired by two tools used to evaluate the usability of information technologies, the *After-Scenario Questionnaire* [25] and *Post-Study Systems Usability Questionnaire* [26]. At the end of the interview, participants were invited to describe the features of an ideal mobile application. A $50 compensation was offered to cover the costs of the shopping purchases and transportation. All interviews were recorded and transcribed verbatim by a collaborator and each transcription was validated/reviewed by the research assistant who conducted the interview. Throughout the project, the student researchers noted in a logbook the observations on the mobile applications’ usability and reported technical problems. The research team and the accessibility expert also tested each of the mobile applications and shared their comments.

#### 2.3.4. Data Analysis of Step 2

The thematic analysis of the transcriptions’ content was completed using NVivo software [27]. The initial coding grid was established from the *After Scenario Questionnaire* [25] by the research assistant and then open coding was used [28]. The research assistant and the co-researcher reviewed the coding and discussed to reach a consensus. The main researcher made a final revision of the coding grid. Also, the research team’s logbook content was analysed to regroup comments concerning content and usability.

## 3. Results

### 3.1. Step 1: Define Users’ Needs

#### 3.1.1. Listing EBF Measuring Tools

**Figure 1:**
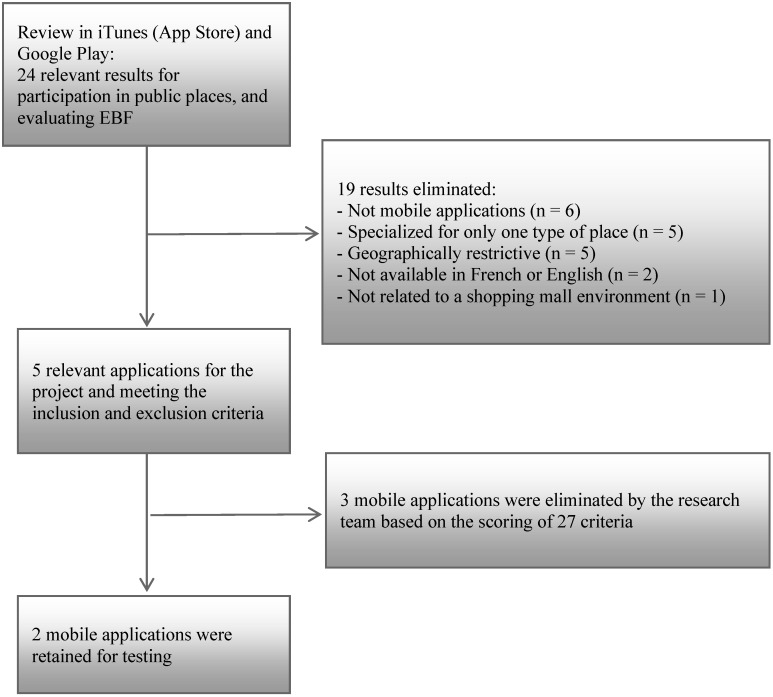
Selection process of the mobile applications.

The review in search engines identified 24 potential mobile applications (see Figure 1). Among these, 19 were eliminated for reasons presented in Figure 1. Five mobile applications met the inclusion and exclusion criteria: *AbleRoad*, *AgeCAP*, *Jaccede*, *Wheelmap*, *Woussoul*.

#### 3.1.2. Content Analysis and Selection of Mobile Applications

*AbleRoad* and *Jaccede* were retained for testing based on the ratings of 27 criteria by the research team.

*AbleRoad* is linked to a web-based consumer guide for the general public called *Yelp*, where consumers share their opinions on all types of service establishments (e.g., rating of food quality in restaurants) [29]. A place must be accessed in *Yelp* to then view its accessibility rating on *AbleRoad*. *AbleRoad* is an American mobile application that allows the user to select his preference in terms of limitation profile (motor, visual, hearing or cognitive limitations). The application allows one to either consult information on places visited by others or to contribute by assessing places using an evaluation grid or by uploading pictures (in several steps). It also includes the identification of technical aids present in the environment. *AbleRoad* offers three research modes: by geolocation (geographical map illustrating results and nearby accessible places), by search engine (types of places in a known city) or by types of disabilities (select preference settings). In addition to a listed display, the results of the research can be displayed as a map. Evaluation criteria (presented for more than 48 items) were established by a committee of experts having accessibility training [30]. Items are rated with a five star ordinal rating scale. It is also possible to make open comments (maximum 24,000 characters) for all of the types of places (e.g., restaurants, food markets). The average scores given by the users are presented in the mobile application and it is possible to access the status of the user who filled in an evaluation (e.g., expert, user with disabilities, family caregiver). *AbleRoad* offers the possibility of judging a previously rated evaluation as helpful or not. It is also linked to social networks (e.g., Facebook, Twitter) and allows sharing or appreciating an evaluation (the mention “Like”). This mobile application is available for free in English on iTunes [31] and GooglePlay [32].

*Jaccede* is linked to a website presenting evaluations of accessible places to encourage and accompany shopkeepers in their process of making their store accessible. Developed in France, *Jaccede* emphasizes environmental facilitators and presents information and icons in a large format. The mobile application allows one to consult information and to contribute by evaluating a place’s accessibility with an evaluation grid with 33 short descriptive evaluation criteria grouped under seven dimensions: description, entrance, interior circulation, services and equipment, other disabilities, environmental accessibility and final notes. The rating system includes a dichotomous scale (yes/no), a nominal scale (e.g., access is complete or partial), an ordinal scale (e.g., bathrooms are fully, partially or not at all accessible) and a ratio scale (e.g., width of the entrance in centimeters). There is also a numerical scale (range 5 to 10) for general accessibility and for the degree to which the environment is welcoming. It allows users unlimited space to add open comments. When the user consults an evaluation on *Jaccede*, only the most recent evaluation is displayed, because the grids of previous reviewers are overwritten. We noticed however, that the initial accessibility score on a 10-point scale remains unchanged. *Jaccede* offers three search engines: by geolocation, by navigation on a map or by place categories. It allows uploading of pictures in an evaluation in a single step. Evaluations are managed by a webmaster (linked to the virtual guide) prior to their posting in the mobile application. This mobile application is freely available in French on iTunes [33] and on Google Play [34].

### 3.2. Step 2: Test Usability

#### 3.2.1. Testing by the Research Team and the Accessibility Expert

The logbook allowed us to collate the comments of the research team and accessibility expert, summarized in Table 2. With respect to the content, the research team and the expert appreciated when the mobile application emphasized the facilitators and technical aids as accessibility criteria. However, they disliked when a mobile application only listed facilitators (e.g., no obstacle appeared in the list of criteria) and thus failed to allow the person to identify obstacles. Some evaluation criteria did not focus enough on the client’s concrete needs (e.g., presence of an elevator, rest area, wheelchair rental) forcing the users to compensate for this limit by adding general comments. The comment section was relevant because it gave users the opportunity to add details to their evaluation. The possibility of uploading pictures, available in both mobile applications, was a function appreciated by the research team and the expert. However, one application required exiting the mobile application to take pictures and then going back to it, lengthening the evaluation time.

**Table 2:** Key features of an ideal mobile application.

Features	Experts *	Users	Examples
***Content***			
**Granularity of the information**		X	Precise, clear and detailed content (e.g., product and paying terminal disposal, floor surface and unevenness, accessibility of fitting rooms)
		X	Description of available products and their location in the store, sales and general information (e.g., address, phone number)
	X		Identification of facilitators, environment’s technical support and barriers to properly represent a location’s accessibility
**Information contextualization**		X	Information on atmosphere, level of client traffic and quality of service
	X	X	Pictures and additionnal comments to ensure conformity between a place’s description and reality
		X	Concrete evaluation criteria
***Usability***			
**Place finding**	X		Geolocation in addition to a variety of research modes (keyboard input, map navigation)
	X		Research based on a personalized profile of wanted facilitators and barriers to avoid
**Rating system**	X		Result presentation in the form of a universally recognized rating (five stars)
	X		Dichotomous scale evaluation grid to do an inventory for all EBF
		X	Precise, clear and objective rating system to describe a place’s physical accessibility
**Presentation of results**	X		Evaluation ratings represented by an average
		X	Results classified by alphabetical order or by type of place
	X		Content presented according to the types of disabilities (mobility, vision, hearing and cognition)
		X	Map maker locates a place and establishes a route
***Usability***			
**Compatibility with targeted clientele**	X	X	Available in French and English
		X	Vocabulary easily understandable
**User-friendliness**		X	Easy to learn
	X		Short and simply formulated items of the evaluation grid
	X	X	Easy to manipulate (e.g., fewer steps to get the information, navigation simplified on one page; pictures uploadable in one step)
	X		Brief evaluation grid to fill
		X	Word prediction when searching for a place
**Aesthetics**	X	X	Content and icons in large format
**Credibility**	X		Identification of the status of the user who filled in the evaluation grid (e.g., date and user’s profile)
	X		Validation by a webmaster (but avoid delays)
**Connectivity or interactiveness**	X		Linked to social network
	X		Associated with a website (consumers’ opinion)
	X		Possibility to judge an evaluation (helpful or not)

Notes: * Includes the researchers, research assistants and an accessibility expert; EBF: environmental barriers and facilitators; X: element mentioned by the group.

The research team and the accessibility expert commented upon several aspects of the mobile applications’ usability. Results presented in the form of a universally recognized rating (five stars) scale was considered to be the most user-friendly because it highlights the strengths and weaknesses of a place with a scale ranging from negative to positive appreciations. The possibility of judging an evaluation (e.g., helpful or not) was appreciated, as well as the display of results in the form of a map. They judged relevant that the covered content was presented for four types of limitations (mobility, vision, hearing and cognition). However, they disliked when an application imposed a minimal accessibility score (e.g., no score under 5 on a 10-point scale is allowed). Moreover, a mobile application that only showed the most recent evaluation was not considered to be representative as it was not possible to see the varied opinions. Short and simple item descriptions were considered facilitatory for people with a mild communication or cognitive limitation. However, evaluations could take a long time to complete and statements were sometimes confusing. In fact, short and simple item descriptions sometimes were insufficient to properly describe an environmental feature. The mobile application’s limited response choices did not always allow nuanced answers, which prevented from adequately representing reality. It was judged beneficial for a mobile application to be associated with a website sharing consumers’ opinions and to be linked to social networks allowing the sharing and appreciation of an evaluation. However, delays associated with the mobile application’s link to the website could considerably slowdown the evaluation process. Indeed, if a place did not appear in the mobile application, there was sometimes a 48-h delay between the request and its inclusion on the mobile application. However, the mandatory review of the evaluation by a webmaster offered further validation, according to the research team and the expert. Even though the verification by the webmaster increased an evaluations’ validity, the delay could cause a feeling of uncertainty (e.g., was the scoring registered?). The research team and the expert appreciated being able to know the status of the user who filled in the evaluation and when information was presented in large format both for the text and the icons. Finally, the language of the mobile application was a significant criterion for the research team and the expert.

#### 3.2.2. Testing by Users

Each testing session was conducted on the same day, for a three to four hour period and generated one screen shot, as well as up to four pictures or videos of EBF per participant. Three users needed help manipulating the tablet when they had to find a store with text entry using the touch pad or taking pictures due to upper extremity functional limitations.

Users evaluated the mobile applications’ content (see Table 2). Some users (n = 3) found the mobile applications’ content was a fair representation of the reality and the accessibility of the stores visited. Users (n = 4) found the information was relevant (e.g., indicates the presence of elevator or stairs, warns that some sections of the store are less accessible). However, some participants (n = 3) reported a gap between the content viewed in the application and actual reality. Users (n = 4) mentioned the content was sometimes vague, incomplete or lacking precision. They would have appreciated detailed and observable evaluation criteria to know what was really evaluated. They would have preferred more information about product disposition in the store (including the payment terminal), the display (e.g., signage, tags), floor surface (e.g., presence of carpet) or unevenness. They also mentioned wanting to be informed of the presence of automatic doors, aisles and stores’ size, fitting rooms’ accessibility, presence of benches, store’s atmosphere (e.g., noise level, luminosity) and the quality of the services (e.g., employees’ attitude and competence, assistance provided, language spoken, availability of delivery services). Participants would have appreciated knowing more about obstacles in the store or having clearly identified non-accessible sections (e.g., bathrooms, rows of cosmetics). Also, a few users (n = 2) found, after having visited a store, that several evaluations were too harsh. A user imagined the impact this could have by saying “[…] I didn’t feel like going and finally it wasn’t that bad. […] If I’d only relied on the evaluation, I wouldn’t have gone to a lot of stores”. Alternately, several participants (n = 3) described the applications as useful, informative, necessary and facilitating because they gave a good idea of the stores’ accessibility. One participant also mentioned that the content of the two tested mobile applications were complementary.

Participants also reported several usability aspects of the mobile applications tested at the shopping mall. Regarding the rating system, the majority of participants (n = 4) found the five star scale lacked precision, clarity and was subjective. One participant, however, appreciated this scale as it gave an overview and allowed a better representation of the store. The nominal scale with descriptive accessibility criteria was appreciated by the participants (n = 4) for its objectivity and precision. Nevertheless, choices were limited in this accessibility nominal scale when it came to evaluating a store. The numerical scale (0 to 10) was not appreciated by a user because it left some leeway for interpretation and was too general. Regarding the application’s design, participants liked having large characters, as these were easier to read and made the application more appealing. The mobile applications were judged easy to control. Clear information facilitated comprehension but the vocabulary was sometimes difficult to understand (n = 4) (e.g., “multi-level access”, “visual guidance disposal”, “threshold”, “sanitary”). Mobile applications were easy to use and to manipulate when the buttons’ size was appropriate and when there was a limited number of steps to obtain the desired information. Participants however found that the way of locating stores with the touch pad made the mobile application more difficult to use, especially when the geolocation function did not work. They found the mobile applications pleasant to use and appreciated the possibility of discovering other evaluated places in the area, as offered by the application menu. Finally, language was an important aspect of the extent to which the mobile application was considered user-friendly for some participants (n = 3) and a French-speaking user said in this regard “…when it is in English, I don’t like it”.

Participants proposed certain features that they would like to find in an ideal mobile application (see Table 2). Four participants would like realistic evaluation criteria providing precise and clear information such as a distinction between the exterior and interior entrances, presence of carpet, fitting room accessibility or presence of stairs in the store. They would like to be informed of the sales and available products, their location in the store, as well as having the store’s general information (e.g., address, phone number). This would allow users to get all the information on the store (including its accessibility) in a single location. Some participants would also like to know the store’s level of client traffic (n = 1), the presence of interior signs (n = 1) and the quality of the service provided (n = 2). These new features for an ideal application would better inform users of a place’s accessibility, specific to their individual needs, prior to them engaging in a shopping activity. Concerning usability, participants (n = 3) would like to use a mobile application that presents the shortest route between stores. Some users would like the ideal mobile application to be able to classify stores by alphabetical order or by type (n = 1), to be available in French (n = 1), and that includes a general comment section (n = 1) such as can be found in two of the tested mobile applications (n = 1). Finally, some would also like to have a word prediction feature when searching places so as to reduce typing time (n = 1). More specifically, as the name of the place is typed, the software would produce a list of words beginning with the letter sequence recorded, and when the target place appears in the list, it could be chosen and inserted into the ongoing text with a single click.

#### 3.2.3. Technical Problems

During the project, various problems occurred with the two tested mobile applications causing some frustration among users, the research team and the expert. First, the developer inadvertently caused the deletion of certain evaluations that had been completed by the accessibility expert when doing system updates, and as a result these evaluations were not available to the users during the testing sessions. This highlights the challenge related to the management of the evaluation database. Some encountered frustration could have been avoided if the user or research team and expert had been informed of certain constraints prior to testing. For instance, users had problems with the 48 h minimum delay imposed to post a new place through the website, or the delay caused by the webmaster’s validation of new evaluation forms; the impossibility of rating lower than 5/10, and the absence of a confirmation that the evaluation was registered. During one testing session, one of the two mobile applications did not work and the user had to consult a binder with photocopied screenshots to simulate the mobile application. Moreover, two users found it particularly difficult to find a store when they had to use the keyboard on the touch pad or when the button size was smaller than the fingertips. These technical problems and functionalities issues possibly had a negative impact on the users’ experience and their perceptions of the mobile application.

## 4. Discussion

The aim of the study was to determine the content and usability features of existing mobile applications assessing the environmental barriers and facilitators to participation (social and physical) in order to respond to the needs of clients with mobility restrictions presenting mild communication or cognitive limitations. The findings from the mobile application testing sessions with users, as well as the comments of the research team and the accessibility expert, generated a list of some 30 important features grouped into 10 categories for an ideal mobile application evaluating EBF. Some features were accepted unanimously such as content and icon presentation in large format, simplicity of navigation (e.g., finding a store), possibility of adding general comments and availability in English and French. However, some important features were differently addressed. First, there was a difference in the perception of the rating system; the research team and the expert appreciated the universally recognized rating system (five stars) while users preferred the nominal scale with descriptive criteria describing a place’s physical accessibility. Regarding content, the users noted more concrete features; the mobile applications’ content was not accurate or objective enough, and some EBF of importance to them were absent (e.g., floor surface and unevenness, benches, automatic doors). Regarding usability, users mostly targeted features that could possibly influence the mobile application’s day-to-day user-friendliness: choice of language, way of locating a store, number of steps to get information, the way information is presented and the type of scale used to evaluate accessibility. In contrast, the research team and the expert prioritized other features of the mobile application having potential influence on its user-friendliness such as its connectivity to an Internet network for sharing evaluations and comments. The new functions proposed (e.g., knowing a store’s level of client traffic, seeing the available products and sales, knowing the quality of the service provided) demonstrate that the mobile applications under study did not meet all of the users’ significant needs.

Involving users is a common practice in studies analysing applicability, usability, content and aesthetics to ensure that a product is appropriate for their needs, is satisfying [35,36] and motivates intention to use [37]. Testing two mobile application options allowed users to compare features and better take ownership of their preferences [38,39]. This collaborative opportunity was perhaps more difficult for some users because of their functional limitations and of the number of tasks requested (e.g., comparing two mobile applications, using the *Photo elicitation* method). Surprisingly, users reported that mobile applications evaluating the EBF could become an obstacle to participation if they evaluated the stores too harshly and thus could diminish a users’ desire to visit the store. *Photo Elicitation* has been used in studies focused on participation in activities of daily living [40,41]. To our knowledge, our project is the first to use this method to test a mobile application. Projects using photo elicitation may vary from a couple of weeks to a few years [42] and the more time participants have to use this method, the more they will use it [43]. In the current study, participants had to use this method for only an hour, which may explain the limited use of this means of expression. We used the *Photo Elicitation* method to encourage dialogue and to help evaluators better understand participants’ perspectives [43], taking into consideration their communication and cognitive limitations. Nevertheless, users hardly used this method during the testing session. Picture-taking with the electronic tablet was a very complex activity (stabilizing the tablet and taking a picture), challenging participants with functional limitations to upper limbs, requiring sometimes the research assistant to take the pictures for them. In the future, it would be interesting to plan an anchoring system to install a digital tablet on a wheelchair to stabilize the device.

This study has certain limits regarding the method of selecting the applications tested in this study, language and communication limitations, and duration of testing. In the absence of users on the selection committee, the research team prioritized the mobile applications’ content, especially variables related to social interactions and the variety of EBF included, at the expense of usability. As a result, users were not exposed to the simplest models of mobile applications with a more limited content, such as a single three-level scale (e.g., *Wheelmap*). Also, the language used by the mobile applications may have represented a bias favoring one mobile application over another (*i.e.*, not for its specific features, but because it was in the user’s mother tongue). Another limitation was that since the enrolled users were not using augmentative and alternative communication devices, our study does not represent the needs of persons with complex communication challenges [4]. Indeed, complex communication challenges are important considerations when developing mobile applications [44]. Finally, the testing over a short period of time represents another limit of the study since it may not have allowed enough time for participants to familiarize themselves with each mobile application, and to get to know its use in daily life. Thus, users did not test all of the possible types of interactions at the shopping mall (e.g., hairdressing salon). Consequently, the varying levels of experience with the mobile technology may have influenced some participants’ ability to comment in a constructive manner.

During the testing sessions, technical problems with the mobile applications occurred (e.g., impossible to find one or several stores), which may have modified participants’ perception of the mobile applications. Also, the mobile applications were updated throughout the testing period, which slightly modified their functionalities (e.g., news feed of the latest evaluation, possibility of uploading pictures in one step, instantaneous evaluations). This may have had an impact on the comments and limited the appreciation of the tested mobile applications.

## 5. Conclusions

The aim of the study was to perform a critical analysis of the content and usability of existing mobile applications as a support tool for persons with mobility restrictions presenting mild communication or cognitive limitations participation, while taking into account the environmental barriers and facilitators for the activities at a shopping mall. This project established a list of desired criteria to inform the development of future mobile applications evaluating environmental barriers and facilitators addressing the needs of the targeted clientele. Eventually, the evaluations captured in these mobile applications could constitute a source of information that would be continuously updated to measure the impact of interventions targeting accessibility awareness in merchants or to monitor the impact of shopping malls’ environmental modifications on the participation of persons with disabilities.

## Appendix: List of Selection Criteria

### User-Friendliness

1. Is the use of the man-machine interface intuitive? (Is it easy to navigate through menus, go back in a selection, *etc.*) ?
2. Does the application have relevant context-sensitive help menus?
3. Does the application have easy to understand context-sensitive help menus?
4. Does the application have at least two search modes of an accessible place (geolocalization/map, place’s address, by type of place, by disability profiles, by types of disabilities/needs, *etc.*)?
5. Does the application have a concern for aesthetics making it appealing and enjoyable to use?

### Content

6. Based on the mapping of the application content, does the application have the minimum content you had expected?

### Psychometric Properties

7. Were the evaluation grids’ psychometric qualities measured empirically or did the evaluation grids come from an instrument whose psychometric qualities were measured empirically?
8. Did the available information on the evaluation grids’ content development (made by whom, when and how) allow attesting its apparent validity?
9. Does the application seem sensitive to change (according to the evaluation method)?
10. Are the items of the evaluation grids formulated in a way to decrease measuring and evaluation errors (to optimize test-retest, inter-rater and intra-rater reliability)?

### Applicability

#### User’s Burden

11. Does the application present the evaluation results in an abbreviated form (e.g., a total score) which is easy to understand?
12. Does the application offer a total score which is an average of the sub-scores given in the evaluation?
13. Is it easy to consult the evaluation grid’s complete details?
14. Does the information provided by the application’s evaluations seem credible (by having, for example a webmaster controlling the evaluation grids, the name of the person who accomplished the evaluation or an option that allows adding pictures supporting the evaluation grids)?

#### User-Evaluator Burden

15. Is the administration time for the evaluation grid reasonable? (a score of 0 can be applied if the administration time seems to take more than 30 min)
16. Does the general formulation of the evaluation grid and items allows its filling without requiring training or specialized vocabulary?
17. Does the evaluation scale and its operational definitions (if present) easy to understand?
18. Are items operationalized and clear (so the evaluator understands without effort the environmental feature he has to evaluate)?

### Compatibility

19. Does the application present a format adapted to one or several disabilities of the following functions?
  - Visual function (e.g., lettering size)
  - Motor function (e.g., button size)
  - Cognitive function (e.g., memory, language, calculation, statement simplification, visual support)
  - Voice and speech function (e.g., voice synthesis, adaptive speech recognition)
20. Is the information contained in the application (e.g., evaluation grids results, instructions) sufficiently precise, clear and adapted to help a user of our targeted clientele’s decision-making?
21. Is the information contained in the application (e.g., evaluation grids results, instructions) sufficiently precise, clear and adapted to help other end-users’ (e.g., merchants, clinicians, family caregivers, researchers, political bodies) decision-making?
22. Is the application available on the Apple and Android systems and on the web?
23. Does the application present the information in French and English?
24. Does the application target adults?

### Other Considerations

25. Is there a large number of places in the area whose accessibility was evaluated and that are listed by the application?
26. Are there strategies put in place to make the application more appealing and easy to use (e.g., a pre-capture of the places and address in the search engines, the application is part of a group of applications for the general public, an alert system informs the user about accessible facilities around him/her) ?
27. Are there marketing strategies put in place to improve the use/popularity of the application (e.g., advertisements, association to an organism of governmental program, visibility by actions)?
